# Outcomes of critically ill coronavirus disease 2019 patients requiring kidney replacement therapy: A retrospective cohort study

**DOI:** 10.3389/fmed.2022.1027586

**Published:** 2022-10-20

**Authors:** Josephine Braunsteiner, Dominik Jarczak, Christian Schmidt-Lauber, Olaf Boenisch, Geraldine de Heer, Christoph Burdelski, Daniel Frings, Barbara Sensen, Axel Nierhaus, Elion Hoxha, Tobias B. Huber, Dominic Wichmann, Stefan Kluge, Marlene Fischer, Kevin Roedl

**Affiliations:** ^1^Department of Intensive Care Medicine, University Medical Center Hamburg-Eppendorf, Hamburg, Germany; ^2^III. Department of Medicine, University Medical Center Hamburg-Eppendorf, Hamburg, Germany

**Keywords:** COVID-19, renal replacement therapy (RRT), AKI, multiple organ failure, ARDS, ECMO, SARS-CoV-2, kidney replacement therapy (KRT)

## Abstract

**Background:**

Coronavirus disease 2019 (COVID-19) has resulted in high hospitalization rates worldwide. Acute kidney injury (AKI) in patients hospitalized for COVID-19 is frequent and associated with disease severity and poor outcome. The aim of this study was to investigate the incidence of kidney replacement therapy (KRT) in critically ill patients with COVID-19 and its implication on outcome.

**Methods:**

We retrospectively analyzed all COVID-19 patients admitted to the Department of Intensive Care Medicine at the University Medical Center Hamburg-Eppendorf (Germany) between 1 March 2020 and 31 July 2021. Demographics, clinical parameters, type of organ support, length of intensive care unit (ICU) stay, mortality and severity scores were assessed.

**Results:**

Three-hundred critically ill patients with COVID-19 were included. The median age of the study population was 61 (IQR 51–71) years and 66% (*n* = 198) were male. 73% (*n* = 219) of patients required invasive mechanical ventilation. Overall, 68% (*n* = 204) of patients suffered from acute respiratory distress syndrome and 30% (*n* = 91) required extracorporeal membrane oxygenation (ECMO). We found that 46% (*n* = 139) of patients required KRT. Septic shock (OR 11.818, 95% CI: 5.941–23.506, *p* < 0.001), higher simplified acute physiology scores (SAPS II) (OR 1.048, 95% CI: 1.014–1.084, *p* = 0.006) and vasopressor therapy (OR 5.475, 95% CI: 1.127–26.589, *p* = 0.035) were independently associated with the initiation of KRT. 61% (*n* = 85) of patients with and 18% (*n* = 29) without KRT died in the ICU (*p* < 0.001). Cox regression found that KRT was independently associated with mortality (HR 2.075, 95% CI: 1.342–3.208, *p* = 0.001) after adjusting for confounders.

**Conclusion:**

Critically ill patients with COVID-19 are at high risk of acute kidney injury with about half of patients requiring KRT. The initiation of KRT was associated with high mortality.

## Introduction

In late 2019 the severe acute respiratory syndrome coronavirus 2 (SARS-CoV-2) emerged and has spread worldwide since then, infecting millions of people (1). The clinical presentation of coronavirus disease 2019 (COVID-19) ranges from mild respiratory symptoms up to severe pneumonia with life-threatening complications, including acute respiratory distress syndrome (ARDS), multi-organ failure and subsequently death (2, 3). Around 20% of patients with SARS-CoV-2 infection have to be admitted to the hospital, and approximately 5% of all patients required treatment in the intensive care unit (ICU) (4–6). While the disease preferentially infects cells in the respiratory tract, there is evidence for involvement of other organ systems, particularly the kidneys (7, 8).

It has been suggested that acute kidney injury (AKI) is associated with COVID-19 disease severity and might be an indicator of poor prognosis (9, 10). Multiple pathogenic mechanisms of COVID-19 associated AKI have been proposed including local inflammation and cytokine release, possible viral invasion, endothelial dysfunction, exposure to nephrotoxins, hypovolemia, coagulopathy, rhabdomyolysis, and impact of mechanical ventilation on renal function (11–13). There is a large heterogeneity in the reported incidence of AKI (7–57%) owing to factors such as variations in clinical management, different definitions of AKI used in clinical research, geographical and socioeconomic differences, pre-existing comorbidities, and severity of disease (14–17). Requirement of kidney replacement therapy (KRT) was frequently reported in about 20–33% of critically ill patients with COVID-19 during the ICU stay (4, 9, 16). Of interest, patients with AKI associated with COVID-19 were more likely to require KRT than those without COVID-19 (16). Additionally, patients requiring both invasive ventilation and dialysis had the highest in-hospital mortality rate of 73% (5). A considerable number of patients with severe COVID-19 suffering from ARDS refractory to conservative management requires veno-venous extracorporeal membrane oxygenation (ECMO) as rescue therapy. In patients with ECMO support a high incidence of AKI (70 to 80%) and KRT was observed and a strong association with mortality was found (18). Currently, there is limited data of risk factors, use and outcome of KRT in critically ill patients with COVID-19. Furthermore, incidence and outcome of KRT in patients receiving ECMO due to severe COVID-19 associated ARDS has not been reported to date.

In the current study we aimed to identify the incidence, risk factors and outcome in critically ill patients with COVID-19 in a large tertiary care center in Germany.

## Materials and methods

### Study population, design, ethics, and primary endpoint

We retrospectively analyzed consecutive COVID-19 patients admitted to the ICU of the Department of Intensive Care Medicine at the University Medical Center Hamburg-Eppendorf (Germany) between 1 March 2020 and 31 July 2021. The study was approved by the Ethics Committee of the Hamburg Chamber of Physicians (No. 2021-300112-WF). Owing to the retrospective character of the study and anonymized data collection, the need for informed consent was waived. The primary endpoint of this study was requirement of KRT.

### Inclusion and exclusion criteria

We included all consecutive adult patients (≥18 years) with confirmed and symptomatic COVID-19. Confirmed COVID-19 was defined as at least one positive result on reverse transcriptase polymerase chain reaction (PCR) obtained from nasopharyngeal swabs and/or bronchial secretions and typical symptoms including dyspnea, fever, or cough. Patients without confirmed COVID-19, ongoing ICU stay at the time of data censoring and patients aged <18 years were excluded.

### Data collection

Patient data was collected from the department’s electronical patient data management system (PDMS; Integrated Care Manager^®^ (ICM), Version 9.1 – Draeger Medical, Luebeck, Germany). The data included positive SARS-CoV-2 PCR, gender, age, body mass index (BMI), comorbidities, admission diagnosis, length of ICU stay, organ support (mechanical ventilation, ECMO, vasopressor support, and KRT), medication, and laboratory test results.

### Clinical definitions and patient management

Severity of illness was evaluated with the sequential organ failure assessment (SOFA) (19) and the simplified acute physiology scores II (SAPS II) (20). The Charlson Comorbidity Index (CCI) (21) was calculated for all patients. Clinical management was performed according to national and international guidelines, including prone positioning in moderate to severe ARDS and, restrictive fluid management following the initial resuscitation period (22). ARDS was defined according to the Berlin definition, using the PaO_2_/FiO_2_ ratio (Horowitz index) as marker for severity (23). Vasopressor support was initiated to maintain a mean arterial pressure (MAP) of 65 mmHg or higher using norepinephrine (22, 24). Patients with severe hypoxemic and/or hypercapnic respiratory failure in combination with severe respiratory acidosis refractory to adjunctive therapies received vv-ECMO. Criteria for the initiation of vv-ECMO support were based on the guidelines of the Extracorporeal Life Support Organization (ELSO) and national recommendations (22, 25). Severe AKI was diagnosed using urine output and/or serum creatinine following the KDIGO guidelines (26). Initiation of KRT followed the most recent Austrian/German recommendations (27, 28). Initiation of KRT was considered by the treating clinician in accordance with local standardized protocols in patients with severe metabolic acidosis (pH <7.2), anuria unresponsive to fluid resuscitation measures, hyperkalemia (serum potassium concentration exceeding 6.5 mmol/L), serum creatinine concentration above 3.4 mg/dl, presence of clinically significant organ edema (e.g., pulmonary edema), or uremic complications (27, 28). KRT in patients with and without vv-ECMO was performed *via* a separate central venous access. Patient survival was assessed at ICU discharge, after 28 and after 90 days. Last day of follow-up was 1 October 2021.

## Statistical analysis

Data are presented as absolute numbers and relative frequency or median with interquartile range (IQR). Categorial variables were compared *via* Chi-square test or Fisher’s exact test, as appropriate. Continuous variables were compared *via* Mann–Whitney-U test. Survival function estimates were calculated using Kaplan–Meier method and were compared by log rank test. To assess factors associated with the requirement of KRT we performed a logistic regression analysis. Clinically relevant variables (age, BMI, gender, septic shock, SAPS II, CCI, ARDS, vasopressors, and ICU length of stay) were included in the initial model and were eliminated stepwise backward. The association between KRT and survival after 90 days was analyzed with a Cox regression model. Clinically relevant variables (age, BMI, gender, ARDS, vasopressors, and KRT) were included in the initial model and were eliminated stepwise backward. We performed an exploratory analysis. Statistical analysis was conducted using IBM SPSS Statistics Version 24.0 (IBM Corp., Armonk, NY, USA). The study protocol was prepared in accordance with the Strengthening the Reporting of Observational studies in Epidemiology (STROBE) recommendations (29).

## Results

### Study population

**FIGURE 1:**
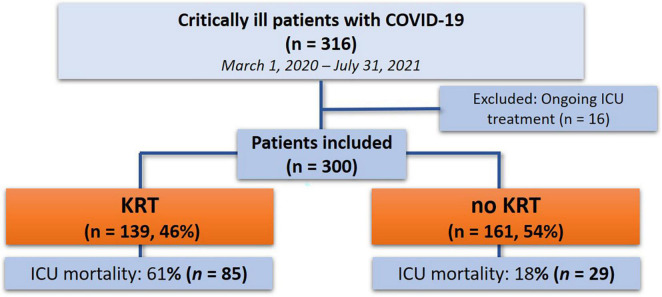
Study flow chart.

Throughout the study period from 1 March 2020 until 31 July 2021, 316 critically ill patients with confirmed SARS-CoV-2 infection were admitted to our department. A total number of 300 patients were included in the study after exclusion of 16 patients with ongoing treatment at the end of the study period (Figure 1). Detailed demographics and baseline characteristics are reported in Table 1 and Supplementary Table 1.

**TABLE 1:** Baseline demographic and clinical characteristics on ICU admission.

Variables	All (*n* = 300)
Age (years)	61 (51–71)
Males	198 (66)
BMI (kg/m^2^)	28.3 (24.7–32.8)
**Disease severity (admission)**	
SAPS II (pts.)	38.5 (32–46)
SOFA (pts.)	8 (3–12)
**Comorbidities**	
Charlson comorbidity index, pts.	1 (0–3)
Arterial hypertension (*n*, %)	156 (52)
Chronic kidney disease (*n*, %)	39 (13)
Coronary heart disease (*n*, %)	41 (14)
Congestive heart failure (*n*, %)	38 (13)
Diabetes mellitus (*n*, %)	93 (31)
Chronic lung disease (*n*, %)	56 (19)
Smoking (*n*, %)	39 (13)
**Admission from**	
Transfer from peripheral ward	87 (29)
Transfer from emergency department	51 (17)
Transfer from other hospital	162 (54)
**Outcome**	
Duration ICU stay (days)	13 (5–29)
Duration hospital stay (days)	25 (11–45)
ICU mortality	114 (38)
28-day mortality	83 (28)
90-day mortality	116 (39)

Data are expressed as *n* (%) or median (interquartile range).

BMI, body mass index; pts., points; SAPS, simplified acute physiology score; SOFA, sequential organ failure assessment; ICU, intensive care unit.

### Occurrence and characteristics of kidney replacement therapy in the study cohort

Of 300 patients, 46% (*n* = 139) required KRT during their ICU stay. Chronic kidney disease prior to ICU admission was observed in 13% (*n* = 39) of the entire cohort. In 40% (56/139) of patients with KRT, KRT was started on the day of admission, in 9 patients KRT had been started in the referring hospital. The median time to start of KRT after ICU admission was 2 (1–8) days and the median duration of KRT on ICU was 13 (4–32) days. Indication for KRT was absolute in 73% (*n* = 102) and relative 27% (*n* = 37). At time of start of KRT there were more than one criterion (absolute or relative indication) present in 65% (*n* = 90) of patients. Primary KRT modality was continuous KRT in 96% (*n* = 133) and intermittent KRT in 4% (*n* = 6) patients. Of patients receiving ECMO (*n* = 91), 70% (*n* = 64) required concomitant KRT. Of patients surviving the ICU stay (*n* = 54) a majority (54%, *n* = 29) were dialysis dependent at time of ICU discharge. For detailed characteristics on indications KRT modality see Table 2. The primary cause of AKI was sepsis/septic shock triggered in 86% (*n* = 119), acute on chronic kidney injury in 12% (*n* = 16) and hemorrhagic shock in 3% (*n* = 4) of patients.

**TABLE 2:** Cause for KRT and modality of KRT.

Variables	KRT (*n* = 139)
**Initiation of KRT**	
Absolute indication	102 (73)
Relative indication	37 (27)
>1 criteria for initiation	90 (65)
Cause for initiation KRT*	
Fluid overload	86 (62)
Fluid overload – present	64 (46)
Fluid overload – prevention	22 (16)
Anuria	54 (39)
Hyperkalemia	62 (45)
Severe metabolic acidosis	68 (49)
– Lactate	49 (35)
Uremia	2 (1)
**KRT modality**	
Primary KRT modality	
CKRT	133 (96)
IKRT	6 (4)
Overall overview of KRT modality	
Continuous veno-venous hemodialysis	130 (94)
Continuous veno-venous hemofiltration	43 (31)
IKRT	24 (17)

Data are expressed as *n* (%) or median (interquartile range). *More than one indication per patient possible.

KRT, kidney replacement therapy; CKRT, continuous kidney replacement therapy; IKRT, intermittent kidney replacement therapy.

### Clinical differences of patients with and without kidney replacement therapy

Clinical characteristics and details on ICU management of patients with and without KRT are reported in Table 3. Critically ill patients requiring KRT were generally older (KRT: median 62 vs. no-KRT: 60 years, *p* = 0.015), had a higher BMI (29.4 vs. 26.9, *p* < 0.001) and were more frequently male (71 vs. 61%, *p* = 0.076) compared to patients without KRT. Comorbidities, represented by the CCI were distributed equally between both groups. Pre-existing immunosuppression was found in 20% (*n* = 23) with and 16% (*n* = 22) patients without KRT (*p* = 0.951). Severity of illness represented by SAPS II (43 vs. 35 points, *p* < 0.001) and SOFA score (11 vs. 5 points, *p* < 0.001) on admission was significantly higher in patients with KRT. Overall, 78% (*n* = 235) of patients received vasopressor support during the ICU stay (86 vs. 61%, *p* < 0.001). In total 73% (*n* = 219) of patients received invasive mechanical ventilation. Placement of ECMO was performed in 46% (*n* = 64) with KRT and 17% (*n* = 27) without KRT in critically ill patients with severe ARDS accompanied by life-threatening hypoxia (*p* < 0.001).

**TABLE 3:** Demographic and clinical characteristics of patients with and without kidney replacement therapy.

Variables	KRT (*n* = 139)	No KRT (*n* = 161)	*p*-Value
Age (years)	62 (55–71)	60 (48–71)	0.015
Males	99 (71)	99 (61)	0.076
BMI (kg/m^2^)	29.4 (25.7–34.7)	26.9 (24.1–31.0)	<0.001
Charlson comorbidity index, pts.	1 (0–3)	1 (0–3)	0.827
**Disease severity**			
SAPS II (pts.)	43 (36–52)	35 (29–41)	<0.001
SOFA – admission (pts.)	11 (7–14)	5 (3–11)	<0.001
SOFA – 24 h (pts.)	12 (8–15)	5 (3–10)	<0.001
**ICU procedures**			
Vasopressors	136 (86)	99 (61)	<0.001
High flow-nasal-cannula	57 (41)	74 (46)	0.957
Non-invasive ventilation	60 (43)	65 (40)	0.587
Mechanical ventilation	131 (94)	88 (55)	<0.001
ECMO	64 (46)	27 (17)	<0.001
**COVID-19 therapy**			
Remdesivir	13 (9)	33 (20)	0.008
Dexamethasone	88 (63)	99 (61)	0.746
Plasma-exchange	4 (3)	3 (2)	0.562
Tocilizumab	3 (2)	7 (4)	0.292
Other antibody-therapy	1 (1)	3 (2)	0.389
**ARDS**			<0.001
No ARDS	11 (8)	85 (53)	
Mild	0 (0)	4 (2)	
Moderate	11 (8)	18 (11)	
Severe	117 (84)	54 (34)	
**ARDS – management**			
Prone positioning	97 (70)	55 (34)	<0.001
Neuromuscular blockade	71 (51)	30 (19)	<0.001
Inhaled vasodilatory treatment	71 (51)	28 (17)	<0.001
Glucocorticoid therapy	120 (86)	104 (65)	<0.001
**Complications – ICU stay**			
Pulmonary embolism	21 (15)	4 (2)	<0.001
Deep vein thrombosis	17 (12)	12 (7)	0.168
Cardiac arrest	31 (22)	14 (9)	0.001
Septic shock	116 (83)	25 (16)	<0.001
Neurologic	37 (27)	37 (23)	0.485
**Urine output, fluid balance, and blood gas**			
Lactate, mmol/L – admission	1.3 (0.9–1.9)	1.1 (0.8–1.7)	0.059
pH, level – admission	7.35 (7.27–7.43)	7.43 (7.36–7.48)	<0.001
Base excess – admission	0.1 (−3.8 to 4.5)	2 (−1.3 to 5.2)	0.021
Bicarbonate – admission	24.1 (21–27.2)	25.9 (23.1–27.9)	0.009
Creatinine, mg/dl – admission	1.47 (0.88–2.85)	0.82 (0.66–1.13)	<0.001
Urine output, ml – day 1	610 (159–978)	1,000 (601–1,674)	<0.001
Fluid balance, ml – day 1	856 (180–2,316)	560 (−156 to 1,158)	0.003
Urine output, ml – day 2	1,110 (248–1,900)	1,800 (1,060–2,700)	<0.001
Fluid balance, ml – day 2	2,573 (634–4,482)	1,125 (200–2,343)	<0.001
Urine output, ml – day 3	940 (88–2,225)	2,200 (1,478–2,950)	<0.001
Fluid balance, ml – day 3	3,242 (1,063–6,222)	1,754 (401–3,166)	0.027
**Outcome**			
Length of stay – ICU (days)	24 (9–43)	9 (3–20)	<0.001
Length of stay – hospital (days)	30 (14–52)	21 (10–34)	<0.001
28-day mortality	55 (40)	28 (17)	<0.001
90-day mortality	84 (60)	32 (20)	<0.001
ICU mortality	85 (61)	29 (18)	<0.001

Data are expressed as *n* (%) or median (interquartile range).

ARDS, acute respiratory distress syndrome; SOFA, sequential organ failure assessment; SAPS II, simplified acute physiology score II; pts., points; ECMO, extracorporeal membrane oxygenation; ICU, intensive care unit.

Complications during the ICU stay were frequent, pulmonary embolism and deep-vein thrombosis were found in 8% (15 vs. 2%, *p* < 0.001) and 10% (12 vs. 7%, *p* = 0.168), respectively. Overall, 15% suffered from cardiac arrest (22 vs. 9%, *p* = 0.001) and 47% from septic shock (83 vs. 16%, *p* < 0.001).

### Kidney function, urine output, fluid balance, and laboratory findings in patients with and without kidney replacement therapy

Detailed information about kidney function, urine output, and fluid balance of patients with and without KRT are reported in Tables 3, 4. Creatinine on admission was 1.47 (0.88–2.85) mg/dl in patients requiring KRT compared to 0.82 (0.66–1.13) in patients without KRT (*p* < 0.001). Further, the median pH level (7.35 vs. 7.43, *p* < 0.001), bicarbonate (24.1 vs. 25.9, *p* = 0.009) and base excess (0.1 vs. 2.0, *p* = 0.021) on admission were lower in patients requiring KRT. On admission and after 24 h inflammatory markers including leukocytes, procalcitonin, interleukin-6, ferritin, and C-reactive protein were significantly higher in patients with KRT (all *p* < 0.001). Further, we observed a significantly higher level of D-dimers on admission and after 24 h in patients with KRT compared to patients without KRT (both *p* < 0.001). Further differences in laboratory parameters on admission and after 24 h in patients with and without KRT can be found in Table 4 and Supplementary Table 2. Urine output during the first 3 days after ICU admission was significantly lower in patients requiring KRT (all *p* < 0.001). Cumulative fluid balance from day 1 to day 3 after admission was significantly higher in patients with KRT.

**TABLE 4:** Laboratory characteristics on admission and after 24 h.

Parameters	KRT (*n* = 139)	No KRT (*n* = 161)	*p*-Value
**Laboratory characteristics – admission**			
Hemoglobin – admission (g/dl)	10.9 (9.5–12.7)	11.7 (10.0–13.0)	0.095
Leukocytes – admission (G/L)	11.5 (7.7–17.1)	8.6 (5.7–13)	<0.001
Thrombocytes – admission (G/L)	242 (160–330)	229 (156–320)	0.336
Creatinine – admission (mg/dl)	1.47 (0.88–2.85)	0.82 (0.66–1.13)	<0.001
Ferritin – admission (μg/L)	1,280 (781–2,392)	834 (372–1,557)	<0.001
MR-pro-Adm – admission (nmol/L)	2.46 (1.46–4.94)	1.19 (0.78–2.12)	<0.001
D-dimer – admission (mg/L)	3.71 (1.7–10.1)	1.91 (0.95–4.43)	<0.001
Lactate dehydrogenase – admission (U/L)	537 (420–659)	417 (344–588)	<0.001
Interleukine-6 – admission (ng/L)	169 (48–525)	52 (16–147)	<0.001
Procalcitonin – admission (μg/L)	0.75 (0.24–3.08)	0.23 (0.08–0.67)	<0.001
C-reactive protein – admission (mg/L)	203 (120–282)	112 (52–210)	<0.001
Bilirubin – admission (mmol/L)	0.8 (0.5–1.2)	0.7 (0.4–1.1)	<0.001
**Laboratory characteristics – day 1**			
Hemoglobin – 24 h (g/dl)	10.2 (9.0–11.8)	11.3 (9.8–12.6)	0.003
Leukocytes – 24 h (G/L)	12.1 (8.0–17.0)	8.5 (5.8–12.4)	<0.001
Thrombocytes – 24 h (G/L)	236 (179–336)	232 (166–314)	0.804
Creatinine – 24 h (mg/dl)	1.52 (0.92–2.47)	0.79 (0.61–1.10)	<0.001
Ferritin – 24 h (μg/L)	1,244 (765–2,491)	792 (264–1,496)	<0.001
MR-pro-ADM – 24 h (nmol/L)	3.21 (1.95–5.89)	1.38 (0.91–2.23)	<0.001
D-dimer – 24 h (mg/L)	4.1 (1.5–9.7)	2.2 (0.9–5.6)	<0.001
Lactate dehydrogenase – 24 h (U/L)	518 (415–624)	401 (302–551)	<0.001
Interleukine-6 – 24 h (ng/L)	155 (46–448)	43 (15–130)	<0.001
Procalcitonin – 24 h (μg/L)	1.32 (0.32–4.72)	0.20 (0.09–0.71)	<0.001
C-reactive protein – 24 h (mg/L)	201 (123–281)	105 (48–196)	<0.001
Bilirubin – 24 h (mmol/L)	1 (0.6–1.7)	0.7 (0.5–1.1)	0.080

*n*, number.

### Risk factors for initiation of kidney replacement therapy

Multivariable regression analysis identified septic shock (OR 11.818, 95% CI: 5.941–23.506, *p* < 0.001), SAPS II (OR 1.048, 95% CI: 1.014–1.084, *p* = 0.006) and vasopressor therapy (OR 5.475, 95% CI: 1.127–26.589, *p* = 0.035) as factors significantly associated with the requirement of KRT initiation (Table 5A).

**TABLE 5A:** Logistic regression model for factors associated with requirement of kidney replacement therapy.

Logistic regression	Covariables	OR (95% CI)	*p*-Value
Final model	Septic shock (yes vs. no)	11.818 (5.941–23.506)	<0.001
	SAPS II (points)	1.048 (1.014–1.084)	0.006
	Vasopressor (yes vs. no)	5.475 (1.127–26.589)	0.035
	ICU – LOS (days)	1.016 (0.998–1.035)	0.074

Hierarchical stepwise backward elimination of insignificant variables, change of parameter estimate >10% = confounding variable. OR, odds ratio; CI, confidence interval; SAPS II, simplified acute physiology score II; ICU, intensive care unit; LOS, length of stay. SAPS II and ICU – LOS – transformation *via* natural logarithm before inclusion into logistic regression analysis.

### Outcome in patients with and without kidney replacement therapy

**FIGURE 2:**
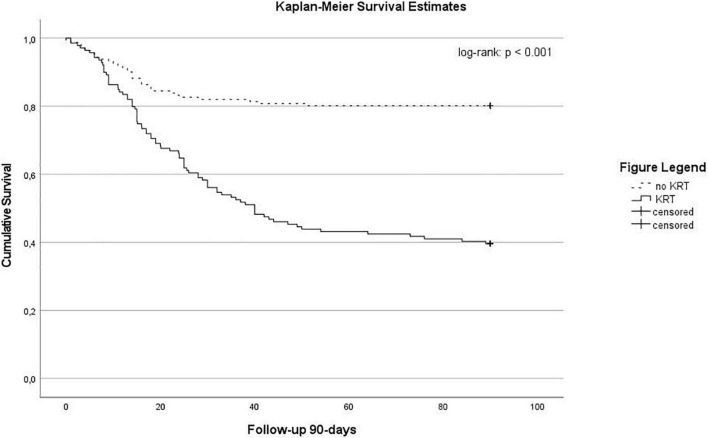
Kaplan-Meier survival estimates stratified according by the use of renal replacement therapy (log-Rank: *p* < 0.001).

The median duration of ICU and hospital stay of patients with and without KRT was 24 (9–43) compared to 9 (3–20) days (*p* < 0.001) and 30 (14–52) compared to 21 (10–34) days (*p* < 0.001), respectively. Overall, a 28-day mortality of 28% (*n* = 83) and 90-day mortality of 39% (*n* = 116) was observed in our cohort. In patients with KRT we observed an ICU mortality of 61% (*n* = 85) compared to 29% (*n* = 18) in patients without KRT (*p* < 0.001). The 28- and 90-day mortality was 40% (*n* = 55) and 60% (*n* = 84) compared to 17% (*n* = 28) and 20% (*n* = 32), respectively (both *p* < 0.001). See also Kaplan–Meier survival estimates for 90-day mortality (Figure 2). In patients with ECMO the ICU mortality was 69% (*n* = 44) in patients with KRT compared to 56% (*n* = 15) in ECMO patients without KRT. Cox regression analysis identified ARDS (HR 4.658, 95% CI: 2.258–9.611, *p* < 0.001), KRT (HR 2.075, 95% CI: 1.342–3.208, *p* = 0.001), and age (HR 1.018, 95% CI: 1.002–1.034, *p* = 0.026) as factors significantly associated with 90-day mortality (see Table 5B).

**TABLE 5B:** Cox regression model for factors associated with 90-day mortality.

Cox regression	Covariables	HR (95% CI)	*p*-Value
Final model	ARDS (yes vs. no)	4.658 (2.258–9.611)	<0.001
	KRT (yes vs. no)	2.075 (1.342–3.208)	0.001
	Age (years)	1.018 (1.002–1.034)	0.026
	BMI (kg/m^2^)	0.975 (0.946–1.006)	0.111

Hierarchical stepwise backward elimination of insignificant variables, change of parameter estimate >10% = confounding variable. HR, hazard ratio; CI, confidence interval; ARDS, acute respiratory distress syndrome; KRT, kidney replacement therapy. Age and BMI were transformed *via* natural logarithm prior inclusion in the cox regression analysis.

## Discussion

In the present study, we investigated the incidence of KRT use in critically ill patients with COVID-19 admitted to a large tertiary care center. We found that almost half of the patients required KRT and initiation was independently associated with mortality. To our knowledge, this is the first study focusing exclusively on clinical characteristics and outcomes of patients requiring KRT in critically ill patients with COVID-19.

### Kidney replacement therapy in coronavirus disease 2019

Requirement of KRT in critically ill patients with COVID-19 was reported in 20–33% (4, 9, 16). Of interest, one study in hospitalized patients showed that patients with COVID-19 were more likely to require KRT than those without (16). Overall, we observed a substantially higher incidence of KRT in our study population compared with previous observations (14). Interestingly the median time to start KRT after admission to ICU was 2 days, which appears quite low. However, this could be attributable to the very dynamic disease course especially in critically ill patients with COVID-19, a high number of septic shock and about one-third of patients requiring vv-ECMO where KRT is often used to facilitate fluid management. However, to date it is unclear if early or late initiation of KRT confers clinical benefits and this question is on an ongoing debate (30). Several factors may account for the higher incidence of KRT in our cohort. First, patients in our study presented with a high disease severity on admission (median SOFA score of 8 on admission). Further, 68% of patients developed ARDS which underlines the COVID-19 related disease burden of our study population. Second, our center is a referral hospital and it is specialized in the treatment of patients with ARDS. Therefore, the illness severity at baseline and the number of patients with multi-organ failure might be higher as compared with other centers. Third, a majority of patients requiring KRT suffered from septic shock and served as an independent predictor for KRT in our cohort. This probably explains the high requirement of KRT and is in line with other studies of patients with sepsis and septic shock (31–34). Of note, the high incidence of KRT may also be an expression of direct kidney involvement, as previously proposed, or complications during the ICU stay like pulmonary embolism, septic shock, or cardiac arrest (8). However, Hardenberg et al. suggest that severe AKI in COVID-19 is tightly intertwined with critical illness and systemic inflammation and is not observed in milder disease courses (7). This might point toward traditional mechanisms of AKI rather than a kidney-specific mechanism (7). Importantly, the incidence of COVID-19-associated AKI seems to be higher compared with other types of severe respiratory failure (35). About 50% of critically ill patients with H1N1 developed AKI that required KRT (16, 36–40). Furthermore, we investigated if the different waves of the pandemic included in this study could have had an impact on the initiation of KRT. We report from three waves of the pandemic in Germany (#1 – 03/2020 to 06/2020, #2 – 07/2020 to 12/2020, and #3 – 01/2021 to 08/2021). We observed numerical differences in KRT initiation, which did not reach statistical significance (49 vs. 45 vs. 46%, *p* = 0.881). We observed that half of the patients who required KRT were dialysis dependent on ICU discharge. This is higher than observed in other previous studies (41, 42). If this is attributable to COVID-19 or probably to an earlier referral to specialized rehabilitation facilities remains unclear and should be addressed in future studies.

### Kidney replacement therapy and mortality in critically ill patients

Patients requiring KRT were significantly older and had a higher BMI, which is in line with previous studies in critically ill patients with COVID-19 (14). The logistic regression analysis identified that septic shock, SAPS II and use of vasopressors are associated with KRT requirement, which underlines the link between initiation of KRT and the severity of illness in the present cohort. Furthermore, patients requiring KRT had a substantially longer stay in the ICU and hospital. In general, the ICU mortality in our cohort was 38%. This is higher than previously reported in Germany (5). Partially, that can be explained by the severely ill population treated and the high number of patients in very critical condition referred to our center. Furthermore, also hemodynamic changes in severely ill patients alongside with mechanical ventilation, vasopressor therapy, and ARDS may be associated with higher KRT risk. We observed a substantially higher ICU mortality in patients with KRT compared to those without KRT. This is in line with several other studies in critically ill COVID-19 patients (43, 44). Further, we could demonstrate that KRT was an independent predictor of mortality in this cohort of critically ill patients with COVID-19.

### Kidney replacement therapy in patients with extracorporeal membrane oxygenation

Extracorporeal membrane oxygenation may be life-saving for patients with severe respiratory failure with potentially reversible causes. Over the past decade, the use of ECMO has increased substantially in ICUs (45). The pooled incidence of AKI and requirement of KRT in patients with ECMO therapy are 63% (AKI) and 45% (KRT), respectively (46). In the subgroup of patients with ECMO, we observed that 70% required KRT. Generally, risk factors for AKI in patients with ECMO are widespread and include older age, pre-existing comorbidities (e.g., cirrhosis), high lactate and, increased bilirubin (47). In patients with ECMO KRT is initiated to manage or prevent fluid overload, followed by AKI and electrolyte disturbances (47, 48). Previously reported 90-day mortality rates of patients with KRT while on ECMO were almost 69%, and the likelihood of dying for patients receiving KRT was reported to be three times higher than that of those without KRT (46). Of interest, we did not find a difference in mortality between ECMO patients with or without KRT. Complications like pulmonary embolism or septic shock that are known risk factors for KRT were significantly more frequent. Those probably concealed potential beneficial effects and lead to a similar outcome in both groups in our cohort. To date, it is unclear whether KRT directly increases mortality risk or whether it merely represents an epiphenomenon of disease severity (47, 49). We furthermore observed a high rate of septic shock and severe ARDS, one patients was placed on VA-ECMO due to cardiogenic shock during the ICU stay.

### Limitations

Our study has several limitations. First, we present the results of a single-center observational study with a high expertise in the management of critically ill patients with COVID-19. Thus, our results may not be generalizable to other cohorts (e.g., less experienced settings, patients with lower illness severity). Second, due to the retrospective design, pre-admission kidney laboratory tests were unknown, and a general sampling of urine, as well as fluid status or kidney sonography was not performed routinely. Third, only requirement of KRT was used to investigate renal failure (severe AKI) which could underestimate the incidence of less severe forms of AKI in this cohort. Further, due to missing pre-hospital data we could not investigate if AKI was community or hospital acquired which probably has an impact on outcome. Generally, our results may not be generalizable to other settings and have to be interpreted with caution. Fourth, we did not investigate the renal recovery during follow-up. This should be addressed in further prospective investigations. Fifth, changes in clinical practice over time may have influenced outcomes of critically ill patients with COVID-19. Sixth, residual confounding is a matter of concern and cannot be entirely excluded.

## Conclusion

In conclusion, we found that critically ill patients with COVID-19 require KRT in about half of cases. Initiation of KRT is associated with high mortality. Septic shock and disease severity serve as independent predictors of KRT requirement. In a subgroup of patients requiring ECMO for refractory respiratory failure survival was independent from the presence of KRT, which warrants further investigation in future larger trials.

## Data availability statement

The original contributions presented in the study are included in the article/Supplementary material, further inquiries can be directed to the corresponding author.

## Ethics statement

The study was approved by the local clinical institutional review board and complies with the Declaration of Helsinki. The study was registered with the Ethics Committee of the Hamburg Chamber of Physicians (No. 2021-300112-WF). Owing to the retrospective character of the study and its anonymized data collection, the need for informed consent was waived.

## Author contributions

JB, KR, and MF participated in study conception and design and contributed to analysis and interpretation of data. JB, DJ, CS-L, OB, GH, CB, DF, BS, AN, EH, TH, SK, DW, MF, and KR were involved in acquisition of data. JB and KR drafted the manuscript. SK, MF, and DW were involved in critical revision of the manuscript for important intellectual content and participated in supervision. All authors read and approved the final manuscript.
