# Supplementary material for: Outcomes of critically ill coronavirus disease 2019 patients requiring kidney replacement therapy: A retrospective cohort study
Source: Front Med (Lausanne). 2022 Oct 20;9:1027586. doi: 10.3389/fmed.2022.1027586 (PMC9630840; doi:10.3389/fmed.2022.1027586)
Supplement: Supplementary file 1 [file Data_Sheet_1.docx]

Supplementary Material

# Supplementary Tables

Supplementary Table 1 – Pre-existing comorbidities

| *Parameters* | *KRT*  *(n = 139)* | *No KRT*  *(n = 161)* | *p*-value |
| --- | --- | --- | --- |
| AIDS *n (%)* | 0 (0) | 0 (0) | 1 |
| Cerebral arterial disease *n (%)* | 16 (12) | 31 (19) | 0.066 |
| Chronic lung disease *n (%)* | 27 (19) | 29 (18) | 0.754 |
| Chronic kidney disease *n (%)* | 26 (19) | 13 (8) | 0.006 |
| Congestive heart disease *n (%)* | 16 (12) | 22 (14) | 0.576 |
| Connective tissue disease *n (%)* | 13 (9) | 6 (4) | 0.046 |
| Coronary heart disease *n (%)* | 19 (14) | 22 (14) | 0.999 |
| Dementia *n (%)* | 2 (1) | 3 (2) | 0.775 |
| Diabetes Mellitus *n (%)* | 48 (35) | 35 (22) | 0.090 |
| Liver cirrhosis *n (%)* | 4 (3) | 4 (2) | 0.519 |
| Peripheral arterial disease *n (%)* | 9 (6) | 3 (2) | 0.042 |
| Malignancy *n (%)*  Solid tumor  Leukemia  Lymphoma  Solid tumor with metastases | 15 (11)  5 (4)  4 (3)  1 (1) | 17 (11)  10 (6)  8 (5)  5 (3) | 0.948  0.300  0.357  0.141 |

*Abbreviations:* AIDS, acquired immune deficiency syndrome; n, number;

Supplementary Table 2 – Blood gas analysis on admission and after 24h

| *Parameters* | *KRT*  *(n = 139)* | *No KRT*  *(n = 161)* | *p*-value |
| --- | --- | --- | --- |
| Blood gas analysis - admission |  |  |  |
| HCO_3-_ - adm. | 24.1 (21 – 27.2) | 25.9 (23.1 – 27.9) | 0.009 |
| Base excess – adm. | 0.1 (-3.8 – 4.5) | 2 (-1.3 – 5.2) | 0.021 |
| pH – adm. | 7.35 (7.27 – 7.43) | 7.43 (7.36 – 7.48) | < 0.001 |
| Lactate – adm. | 1.3 (0.9 – 1.9) | 1.1 (0.8 – 1.7) | 0.059 |
| paO_2_ – adm. | 72.9 (63.9 – 91.6) | 77.4 (63.9 – 98.1) | 0.414 |
| paCO_2_ – adm. | 45.6 (34.9 – 60.2) | 39.6 (33.0 – 47.3) | 0.001 |
| Blood gas analysis – day 1 |  |  |  |
| HCO_3-_ – 24h | 24.5 (22.2 – 28.2) | 26.0 (23.7 – 28.8) | 0.016 |
| Base excess – 24h | 0.9 (-2.9 – 4.8) | 2.0 (-1.0 – 5.6) | 0.075 |
| pH – 24h | 7.39 (7.31 – 7.43) | 7.44 (7.39 – 7.47) | < 0.001 |
| Lactate – 24h | 1.7 (1.2 – 2.4) | 1.2 (0.9 – 1.8) | < 0.001 |
| paO_2_ – 24h | 72.6 (65.4 – 81.7) | 71.7 (64.2 – 81.9) | 0.650 |
| paCO_2_ – 24h | 46.4 (38.4 – 55.6) | 38.5 (33.8 – 46.3) | < 0.001 |

*Abbreviations:* n, number;
